# Nanoencapsulation of Asparagus ( *Asparagus officinalis* L.) Extract in Nanoliposomes: Effects on Physicochemical, Antioxidant, and Sensory Properties of Functional Processed Cheese

**DOI:** 10.1002/fsn3.70284

**Published:** 2025-05-14

**Authors:** Parisa Solhi, Mahdieh Salari, Hamed Hamishehkar

**Affiliations:** ^1^ Department of Food Science and Technology, Faculty of Agriculture University of Tabriz Tabriz Iran; ^2^ Drug Applied Research Center Tabriz University of Medical Sciences Tabriz Iran

**Keywords:** antioxidant activity, functional foods, nanoencapsulation, natural plant extract, physicochemical properties

## Abstract

In this study, thin‐layer hydration combined with high‐shear homogenization and sonication was used for asparagus ( *Asparagus officinalis* L.) extract loaded nanoliposomes (AENL) preparation. Then its characteristics, including encapsulation efficiency, particle size, zeta potential, and particle shape, were investigated. In the next step, asparagus extract (AE) (in both free and nanoliposome form) was added to processed cheese (PC) formulation at levels of 0 (control), 2%, 10%, and 20% w/w. Cheese samples were kept at 4°C for 60 days, and their pH, acidity, total phenolic content, and antioxidant activity were analyzed. The obtained results showed that the AE‐loaded nanoliposomes had relatively high encapsulation efficiency (68%). The zeta potential of the asparagus extract loaded nanoliposomes solution was −2.09 mV. Regarding the particle size based on the number, most of the particles were below 100 nm, and the average particle size and multiple dispersion index were 151.7 ± 2.11 nm and 0.535 ± 0.019 nm, respectively. Photographs obtained by TEM showed nano‐sized spherical bilayer liposomes without aggregation. Cheese samples containing AE and AENL had higher pH and lower acidity than the control sample (*p* < 0.05). TPC and antioxidant activity of PC samples decreased during 2 months of storage, although this decrease was significantly lower for cheese samples containing encapsulated extract than for other samples (*p* < 0.05). AENL did not have an adverse effect on the sensory properties of fortified cheese. In general, it can be said that AENL, especially at a concentration of 10%, can be successfully used to produce functional cheese.

## Graphical abstract

Thin‐layer hydration combined with high‐shear homogenization and sonication was used for AENL preparation. Nanoencapsulation increased the stability of bioactive and phenolic compounds of AE. AENL was successfully used for the enrichment of processed cheese. Nanoliposomes had no adverse effect on sensory properties of processed cheese.

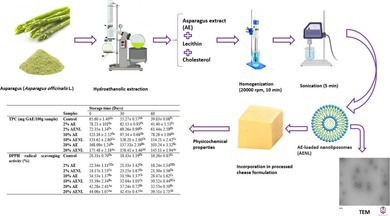

## 1 Introduction

In recent years, the use of bioactive food compounds in the functional foods production has increased dramatically. Functional foods, which offer health benefits that extend beyond basic nutritional value, can play a substantial role in improving overall health (Abedinia et al. 2025). Due to the sensitivity of bioactive compounds to environmental and process conditions (such as oxygen, high temperature, light, pH changes, etc.) and also to mask unpleasant taste and flavor, control the release rate, and precise delivery to the human body, encapsulation technology can be a promising and suitable solution for realizing these objectives (Arpagaus et al. 2024; Gorzin et al. 2024). Encapsulation can improve stability in humid and high‐temperature environments, resulting in long‐term release of bioactive food substances. In addition to the above, using this technology can minimize unwanted and adverse chemical reactions with other food ingredients (Singh et al. 2023).

With the advent of nanotechnology and its potential benefits, researchers have sought to develop nano‐based techniques and materials in the food industry. Food bioactive compounds can be loaded into nanocarriers and nanoparticles and used as unique nano‐delivery systems to produce food delivery systems and functional foods (Arpagaus et al. 2024). Among the nanoencapsulation systems, lipid nanoparticles and nanoemulsions seem appropriate for food requests. Nanoemulsions are very small and kinetically stable emulsions and consist of lipid droplets with nanometric size (50–200 nm), which are dispersed in the aqueous phase and homogenized using high pressure and a sufficient emulsifier (Schultz et al. 2004). Liposomes are vesicles that have a spherical structure and are made of two‐layer membranes (primarily phospholipids) and are applied as a delivery system to encapsulate bioactive compounds such as antimicrobial substances, flavor compounds, and enzymes, etc. (Zhao et al. 2024). Unlike conventional emulsions, nanoemulsions exhibit higher resistance and stability against gravitational separation and aggregation due to their high surface‐to‐volume ratio (Zabot et al. 2022).

Plant extracts are rich in bioactive compounds capable of suppressing microbial growth and demonstrating notable antioxidant properties (Esmaeili et al. 2024). Asparagus ( *Asparagus officinalis* L.) or the “king of vegetables”, is very nutritious and contains a variety of bioactive compounds comprising polyphenols (mainly hydroxycinnamic acids and flavonoids), saponins, anthocyanins, dietary fiber, organic acids, minerals, vitamins, polysaccharides and it covers oligosaccharides such as inulin. Asparagus has a wide range of health benefits, such as antitumor, antimicrobial, neuroprotective, anti‐tyrosinase, anti‐inflammatory, and antioxidant properties (Grohar et al. 2024; Xiao et al. 2025). Among bioactive components existing in asparagus, inulin is a fructooligosaccharide (FOS) and is known as nondigestible dietary fiber. Therefore, due to the high degree of polymerization, it can play a prebiotic role that stimulates the growth of intestinal microbiota. In addition to its prebiotic effect, inulin plays a role in the absorption of mineral ions from the intestine, lipid metabolism, obesity prevention, and blood sugar regulation (Singh et al. 2017). Therefore, it can be a suitable option for fortification of food products.

Processed cheese is produced by blending natural cheeses and other dairy and nondairy products, including whey powder, butter, water, proteins, and vegetable fats in the presence of emulsifying salts and additives (flavors, colors, stabilizers, sweetening, and acidifying agents) via heating and mixing (Deshwal et al. 2023). The advantages of this type of cheese include less need for refrigeration and higher stability. Fortification of processed cheese offers a new functional product with different taste and flavor, thereby enhancing health benefits in addition to increasing consumer acceptance. The main methods used to enrich processed cheese include adding prebiotics and probiotics, vitamins, and other macronutrients (Talbot‐Walsh et al. 2018).

In a previous study on processed cheese fortified with asparagus powder, the antioxidant activity and phenolic content of cheese samples decreased during storage, which explained that the absorption of phenolic compounds by cheese peptides could cause this decrease (Solhi et al. 2020). These peptides can be produced by the residual proteolytic agents during storage, which deactivate the phenolic compounds present in cheese (Fadavi and Beglaryan 2015). In general, proteins and peptides inherently contain reactive sites that are prone to interactions with phenolic compounds, resulting in protein/peptide–phenol binding (Hamzalioglu et al. 2023). Polyphenols with larger molecular weights and those possessing more hydroxyl or galloyl groups provide increased opportunities for interaction with proteins due to their greater number of binding sites (Pérez‐Gregorio et al. 2020). Therefore, this research aimed to investigate the effect of nanoencapsulation of aqueous asparagus extract on the stability of antioxidant activity and phenolic content of processed cheese by preventing unwanted interactions between peptides and phenolics.

## 2 Materials and Methods

### 2.1 Materials

Egg lecithin (Highly purified, CAS 8002‐43‐5), cholesterol (Highly purified, CAS 57‐88‐5), Tween 80 (CAS 9005‐65‐6), ethanol (96%, CAS 64‐17‐5), Folin‐Ciocalteu reagent (CAS 12111‐13‐6), 2,2‐diphenyl‐1‐picrylhydrazyl (DPPH, CAS 1898‐66‐4), sodium carbonate (CAS 497‐19‐8), and potassium hydroxide (CAS 1310‐58‐3) were purchased from Merck Company (Darmstadt, Germany). Trisodium citrate (CAS 68‐04‐2) and disodium phosphate (CAS 7558‐79‐4) were obtained from Sigma Aldrich Chemical Co., Germany. Feta cheese and butter were provided by Kalleh Dairy Co., Iran. All chemicals were analytical grade.

### 2.2 Extract Preparation

Fresh asparagus ( *Asparagus officinalis* L.) was purchased from a supermarket (Tabriz, Iran). The herbarium specimens were preserved in the Pharmacognosy Department of the Faculty of Pharmacy at Tabriz University of Medical Sciences under code 401. Asparagus samples were kept in an incubator (35°C ± 0.5°C) to dry and reach constant weight. The dried samples were then ground (≈40 mesh). Next, 1 ± 0.01 g of asparagus powder was added to 30 ± 1 mL of aqueous ethanol solution (80% ethanol) and stirred (450 rpm) for 60 ± 1 min at 25°C ± 0.5°C. The resulting solution was filtered through Whatman No. 4 filter paper, and the extraction procedure was repeated again. A vacuum rotary evaporator (Heidolph Instruments Co., VV 2000, Germany) at 40°C ± 0.5°C was used to recover the solvent. The final extract was dried with a freeze‐dryer (Christ, Germany) (Vieira et al. 2020). Determination of maximum absorbance was performed by evaluating different dilutions of asparagus extract using a spectrophotometer (Pharmacia Biotech Co., England).

### 2.3 Preparation of Encapsulated Asparagus Extract With Liposome

Thin film hydration‐sonication method was used to prepare liposomes. Lecithin and cholesterol (50:10 w/w) were mixed in 15 ± 0.5 mL ethanol in the presence of 2–3 drops of tween 80 in a round flask. The solvent was evaporated using a rotary evaporator under reduced pressure at 40°C ± 0.5°C, then 5 ± 0.1 mL of asparagus extract was added to the flask and shaken. Next, 5 ± 0.1 mL of distilled water was gradually added and shaken by vortex. For size reduction of nanoparticles, homogenization (Heidolph Silent Crusher M, Germany) with 20,000 rpm for 10 ± 1 min and probe sonication (Heidolph, Schwabach, Germany) set as sequence pulsing of 1 min on and 1 s rest at an amplitude of 40% for 5 ± 1 min, were applied (Xia and Xu 2005).

### 2.4 AENL Analysis

#### 2.4.1 Encapsulation Efficiency (EE)

To calculate the encapsulation efficiency, 1 ± 0.05 mL of the obtained nanosystem was diluted with 1 ± 0.05 mL of distilled water and placed in a Millipore Amicon Ultra filtration tube (Ultracel, 30 kDa cutoff) and centrifuged at 3000 × *g* for 5 ± 1 min. The amount of free extract in the lower chamber was monitored spectrophotometrically (Pharmacia Biotech Co., England, ʎmax =340 nm) and, the encapsulation efficiency was calculated according to the (Equation 1) (Sarabandi et al. 2019):

(1) $$\mathrm{EE}\;\left(\%\right)=W\;\left(\text{added extract}\right)-W\;\left(\text{free extract}\right)/W\;\left(\text{added extract}\right)\times 100$$

W (added extract) is the amount of extract added during the preparation of liposomes and W (free extract) is the amount of free extract measured in the lower chamber of the Millipore Amicon after centrifugation.

#### 2.4.2 Particle Size and Zeta Potential

After the preparation of nano complex solutions, particle size and zeta potential were analyzed using dynamic light scattering (DLS) and zeta sizer (Malvern Instruments Ltd., UK) at a temperature of 25°C ± 0.1°C (Zeta Sizer, Malvern Instrument, Malvern, UK) (Soltanzadeh et al. 2021). The average particle size was determined based on the volume, number, and intensity mean diameter. On most particles in contact with the liquid, an electric charge is created, called zeta potential. Zeta potential indicates the stability of colloidal suspensions or emulsions. Therefore, the higher the zeta potential, the more stable the suspension.

#### 2.4.3 Transmission Electron Microscopy (TEM)

Shapes and layers of particles were evaluated via Transmission electron microscopy (Zeiss‐Leo 906 TEM, Germany) at 150 kV by the method of Wang et al. (2023). In brief, the liposome solution (10 ± 0.2 mL) was diluted with distilled water, deposited on the copper grid with carbon film, and stained using sodium phosphotungstate (1%). Before imaging, the samples were dried at ambient temperature.

### 2.5 Processed Cheese Preparation

Processed cheese samples were prepared according to the method of Solhi et al. (2020). The fortified and control PC samples were manufactured in a vertical vacuum mixer (Stephan Machinery Corp., Mundelein, IL, USA). The PC included a mixture of Feta cheese, butter (3%), water (5%), emulsifying salts (2%) (trisodium citrate (E331) (1%), and disodium phosphate (E339) (1%)) and different levels of asparagus extract in both free (AE) and nanoliposome (AENL) form (0% (control sample), 2%, 10%, and 20%), which was mixed (250 ± 2 g) and melted at 85°C ± 0.5°C for 4 min. The molten cheese samples then cooled to 4°C ± 0.5°C and were kept in a refrigerator for further analysis.

### 2.6 AE‐ and AENL‐Fortified Processed Cheese Analysis

#### 2.6.1 Acidity and pH

The titratable acidity (TA) of the cheese samples was analyzed by the method as described in AOAC (2007), using (Equation 2). The pH value was measured using a digital pH meter (PHC3031‐9; Radiometer Analytical, Copenhagen, Denmark), which was fitted with a glass electrode.

(2) $$\mathrm{TA}\%=\frac{V\times N\times 0.09}{W}$$

where *V* is the volume of NaOH used (mL), *N* is the normality of NaOH (0.1 N), 0.09 is the milliequivalent weight of lactic acid (g/mEq) and *w* is the weight of the cheese sample (g).

#### 2.6.2 Total Phenolic Content (TPC)

The method described by Apostolidis et al. (2007) was used to measure phenol. A test tube was filled with homogenized processed cheese water extract (PCWE, 1 ± 0.05 mL) and mixed with distilled water (5 ± 0.2 mL) and ethanol (1 ± 0.05 mL). In the next step, Folin–Ciocalteu reagent (0.5 ± 0.01 mL, 50% (v/v)) was added to each test tube and mixed. The PC samples left to stand for 5 min and then were added Na_2_CO_3_ (1 ± 0.05 mL, 5%), followed by standing for 1 h. A UV–Vis spectrophotometer (Pharmacia Biotech Co., England, 725 nm) was applied to determine absorbance. Different concentrations of gallic acid were used to create standard curves, and the results obtained were expressed as mg gallic acid equivalent (GAE) per 100 g of PC sample.

#### 2.6.3 Antioxidant Activity

The DPPH radical scavenging activity was determined according to the procedure explained by Yang (2024). For this purpose, 20 ± 1 μL of PCWE sample was mixed with 180 ± 1 μL ethanol solution that contained DPPH radical (0.01%). The resulting mixture was shaken well and kept in dark conditions (25°C ± 0.5°C) for 1 h. Finally, the absorbance of the samples was calculated at 517 nm against blank. The results were calculated using (Equation 3):

(3) $$\text{Inhibition}\;\left(\%\right)=\frac{A_{C}-A_{S}}{A_{C}}\times 100$$

A_C_ and A_S_ are the absorbance of the blank and samples, respectively.

#### 2.6.4 Sensory Analysis

Sensory characteristics of cheese samples were evaluated in terms of odor, color, flavor, texture, and overall acceptance by 14 trained panelists (seven women and seven men, age: 22–40 years). All samples were of equal weight and were assigned random three‐digit codes. Each cheese sample was then poured into a plastic tray and presented to the participants in a room. The analysis was based on a 5‐point hedonic scale (1–5, from very unacceptable to very acceptable).

### 2.7 Data Analysis

SPSS statistical software (IBM SPSS Statistics 22) was used to analyze data by one‐way analysis of variance. All means of the main effect were analyzed with Duncan's HSD test, and the significance level was set at *p* < 0.05. All experiments were done in triplicate.

## 3 Results and Discussion

### 3.1 AENL Characterization

#### 3.1.1 Encapsulation Efficiency (EE)

The encapsulation efficiency indicates the percentage of the bioactive compound, which is the ratio between the initial amount of extract and the amount of free and unencapsulated extract in the formulation. Therefore, the higher the value of the parameter EE, the more phospholipids can protect the bioactive compounds against harmful external factors. Accordingly, the bioavailability of the bioactive compounds will increase (Tungadi 2024). In order to evaluate the encapsulation efficiency, the maximum absorbance of different dilutions of asparagus extract was obtained by spectrophotometer at a wavelength of 340 nm (maximum 340 nm). The encapsulation efficiency of asparagus extract was 68% and appears to be suitable. In a study conducted on linseed oil encapsulated by spray drying using different combinations of wall materials, the researchers stated that the results obtained for encapsulation efficiency could not be related to droplet size or emulsion viscosity and were probably due to the polymer matrices (Carneiro et al. 2013). According to the study of Barbosa et al. (2005), encapsulation efficiency increases with increasing emulsion stability; that is, the amount of non‐encapsulated material on the particle surface is lower. Charve and Reineccius (2009) obtained a similar result by studying the retention of volatiles in microcapsules prepared by spray drying, in that modified starch caused more oil retention in comparison to Arabic gum and whey protein. In the study conducted by Takma et al. (2023), the impact of nanoparticle production techniques and process parameters on the encapsulation efficiency (EE) of zein nanoparticles was examined. The findings revealed that the EE values for the homogenization and nanoprecipitation methods were relatively similar and significantly higher than those achieved using the ultrasonic method. Both the concentration of zein and, particularly, the extract ratio had a notable influence on the EE of the nanoparticles. Additionally, as the extract ratio increased, a significant decline in the EE of the nanoparticles was observed. It has been reported that although liposomes exhibit several advantages such as long‐term efficacy, excellent biocompatibility, and targeted delivery, the encapsulation efficiency is low (Yu et al. 2025). In this regard, Chanioti et al. (2021) stated that the use of nano‐scale encapsulation not only improves the stability and efficiency of droplets, but also increases solubility, prevents chemical reactions, regulates the speed of digestion and absorption in the digestive system, and controlled release; which is due to their high ratio of area to volume. Therefore, they show entirely different physicochemical behavior compared to micro‐ and macro‐scales.

The properties of bioactive agents are influenced by variable factors in the preparation process of liposomes, such as the volume of the aqueous phase, the stiffness of the membrane, the surface area, and the preparation method (Ghorbanzade et al. 2017). EE related to hydrophilic compounds such as betanin‐nanoliposomes (Amjadi et al. 2018), anthocyanin (Kar et al. 2019), *Centella Asiatica* leaf extract (Tripathy and Srivastav 2023), and L‐carnosine (Maherani et al. 2012) were calculated as 80.35%, 77.12%, 40%, and 17%, respectively, in which the EE related to betanin is more suitable and has higher acceptability due to betanin ionic adsorption to the inner and outer surface of nanoliposomes accompanied by incorporating the inner aqueous phase. In the case of phytosomal structures, high values of EE have also been reported, which could be because of this (Babazadeh et al. 2017).

#### 3.1.2 Zeta Potential

**FIGURE 1:**
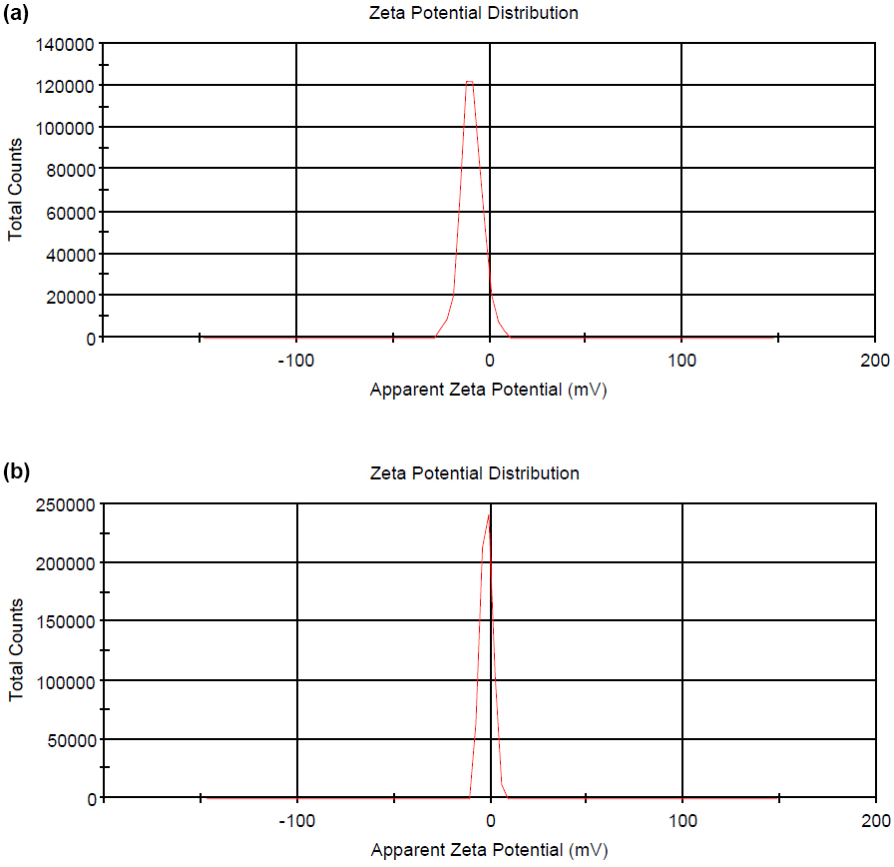
Zeta potential of blank solution without asparagus extract (AE, a) and asparagus extract‐loaded nanoliposomes solution (AENL, b).

To evaluate the electrochemical equilibrium at the interface of nanoparticles, zeta potential was analyzed. Zeta potential indicates the stability of liposomes. Therefore, its calculation is valuable for controlling the sedimentation and aggregation of nanoliposomes, which are parameters that influence physical stability. The zeta potential depends on various factors, including the adsorbed layers on the surface, the surface charge of the vesicles, and the essence of the medium used (Tavakoli et al. 2018). According to the curve of zeta potential achieved by the DLS method, the blank sample (nanoparticles without any asparagus extract) has a higher negative zeta potential (−9.30 mV) than nanoparticles containing asparagus extract (−2.09 mV) (Figure 1). The zeta potential of nanoparticles containing asparagus extract is closer to zero, representing those positive charges existing in asparagus extract neutralize the negative charges of nanoliposomes. In addition, it is possible that during the encapsulation of the extract, in addition to the nanoliposome, nanophytosome was also produced, which reduced the negative zeta potential. However, the zeta potential can be adjusted by adsorbing selected materials onto the surface of the produced nanoparticles and by changing the pH of the medium (Feng et al. 2020). In agreement with our study, Liu et al. (2024) reported that the surface charge of liposomes changes after the addition of carotenoid extract. In colloidal systems, the surface charge of particles (like liposomes) is a function of several parameters, including temperature, ionic strength of the medium, concentration and type of stabilizer, concentration and type of active compound, and concentration and kind of phospholipid (Siyar et al. 2022). In addition, it has been reported that by reducing the size of particles (in nano‐scale) and increasing the ratio of surface to volume, charge density and zeta potential of particle surfaces should increase. As a result, the electrostatic repulsion between liposomes increases, which leads to improved stability and reduced aggregation (Parhizkary et al. 2024).

Zeta potential has a significant impact on particle stability and represents the potential difference between the stabilized and dynamic ionic layers on charged particles. High negative or positive zeta potential causes strong repulsion and a more stable colloidal system (Shamsara et al. 2015; Soltanzadeh et al. 2021). The accumulation of nanoparticles in the oral delivery system is undesirable because it can lead to the uncontrolled release of bioactive compounds (Muhammad et al. 2020).

#### 3.1.3 Size Distribution

Among the characteristics indicating the stability of nanoliposomes, size distribution plays a major role. Polydispersity is defined as an index for the diameter distribution of particles in colloidal systems. Uniformity in diameter is expressed by narrow particle diameter distribution and low polydispersity index (PDI). In other words, the lower the value of the PDI index, the similarity between the nanoliposomes sizes is more remarkable and they have a more uniform distribution. As a result, they exhibit higher stability (Aboutalebzadeh et al. 2022).

**FIGURE 2:**
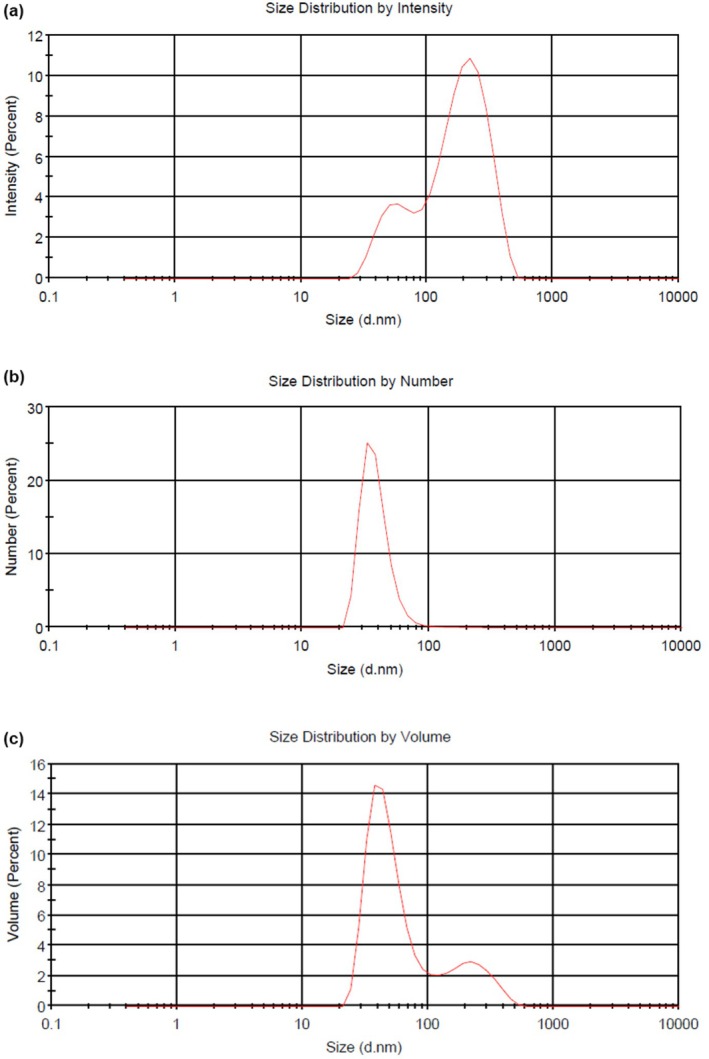
Size distribution of asparagus extract loaded nanoliposomes (AENL) according to intensity (a), number (b), and volume (c).

According to the particle size based on number, some particles were below 100 nm (Figure 2a), and the average particle size and PDI were 151.7 ± 2.11 nm and 0.535 ± 0.019 nm, respectively (Table 1). Refai et al. (2017) reported that a PDI value below 0.7 nm is acceptable for liposomes, indicating proper particle homogeneity. The use of various nonionic surfactants, stabilizers, and phospholipid concentration is the most critical parameter affecting the size of nanoliposomes (Parhizkary et al. 2024). The addition of extract can significantly affect the size distribution of nanoliposomes. Al‐subhi (2025), in a study on nanoencapsulation of yellow *Capsicum Annuum* extract (YCAE) using whey protein isolate (WPI), found that with the addition of YCAE and increasing its concentration, the particle size and PDI of nanoparticles increase. Similar results were obtained by (Soliman, Karam‐Allah, et al. 2024). Reducing the PDI and particle size can affect the viscosity, particle stability, creaminess, appearance, and color of nanoemulsions. It has been stated that as the droplet size decreases, the effect of Brownian motion and gravitational force decreases. As a result, the probability of their joining and coagulation is significantly reduced. It is essential to mention that the enlargement and accumulation of droplets are among the most critical factors in the instability of emulsions (Aboutalebzadeh et al. 2022). Figure 2b,c shows the size distribution of nanoparticles of asparagus extract obtained from the particle size device, respectively, in number mean diameter (NMD) and volume (VMD). The slight difference between VMD and NMD indicates a uniform distribution of prepared nanoliposomes. Cholesterol plays a role in the structure of the lipid carrier and the orientation of the head groups in the membrane interface regions. According to the study of Mohammadi et al. (2014), different amounts of cholesterol were evaluated in vitamin‐containing liposomes, and no significant change was observed in the size and size distribution of vesicles. Cholesterol is placed in the cavities formed by surfactant monomers, which are collected in vesicles inside the liposome layer.

**TABLE 1:** Encapsulation efficiency, particle size, and PDI index of asparagus extract loaded nanoliposomes (AENL).

Sample	Encapsulation efficiency (%)	Particle size (nm)	PDI index
AENL	68	151.7 ± 2.11	0.535 ± 0.019

#### 3.1.4 Morphology

**FIGURE 3:**
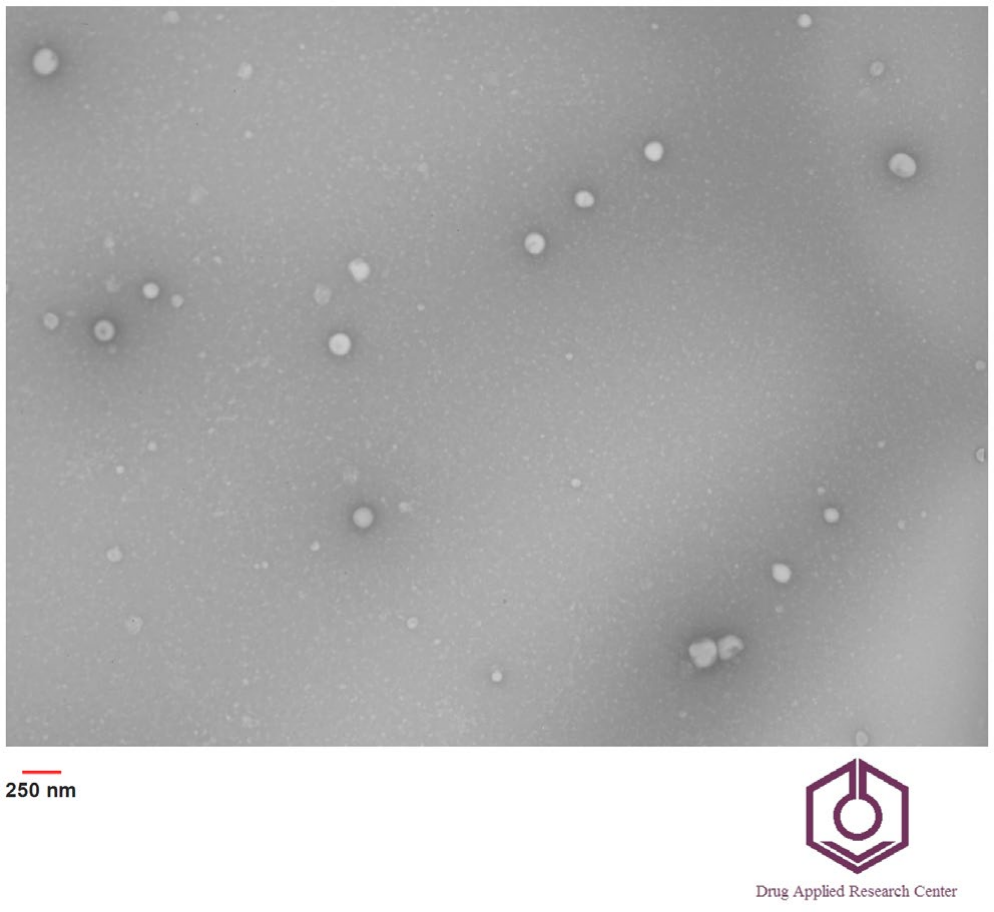
TEM micrograph of asparagus extract loaded nanoliposomes (AENL).

TEM photographs were used to observe the size, uniformity, integrity, and shape of nanoliposomes. Bilayered, spherical, homogeneous, and small liposomes were observed without aggregation, deformation, or leakage (Figure 3). Kuznetcova et al. (2020) stated that the presence of darker and lighter areas in the TEM images of vesicles indicates their multilayered structure. In addition, they had an average diameter of 120 nm and a uniform size distribution, confirming that the AENL achieved the desired nanoscale size. This phenomenon can be due to the effect of the high mechanical strength of the ultrasound treatment used in the preparation of nanoliposomes, which leads to the improvement of the uniformity of the size distribution and the reduction of the size of the vesicles (Pan et al. 2018). These findings are entirely aligned with the dynamic light scattering (DLS) data, which suggest that the prepared nanoliposomes exhibit a favorable dispersion index and homogeneity (section 3.1.3). The samples analyzed by TEM were air‐dried, resulting in the removal of water, whereas the zeta sizer measurements reflected the hydrodynamic diameter of the nanoliposomes, including a surrounding layer of water. Consequently, the TEM images of AENL displayed a smaller size compared to the DLS data. Similar results were reported by Soliman, Karam‐Allah, et al. (2024).

### 3.2 AE‐ and AENL‐Fortified Processed Cheese Characterization

#### 3.2.1 Acidity and pH

Table 2 shows titratable acidity and pH values of PC samples containing different concentrations of AE and AENL during 2 months of storage at 4°C. By increasing the concentration of AE and AENL loaded nanoliposomes to processed cheese, the pH of the samples increased and their acidity decreased. This could be due to the presence of distilled water in the formulation of the extract and liposomes, ultimately diluting the processed cheese and approaching the pH of distilled water, as well as the materials used to produce liposomes, such as lecithin, which have a pH of around 7. On the other hand, throughout 2 months of storage, a continuous increase and decrease of acidity and pH were observed for all fortified and control cheese samples, respectively. The phenolic compounds present in the extract can undergo oxidative hydrolysis, breaking down into phenolic acids with varying acid strengths. This process raises the acidity of the cheese and results in a decrease in pH (Mousavi et al. 2023). In addition, the production of lactate and the partial decomposition of sugars by microorganisms can also decrease the pH and increase the acidity of cheese samples during cheese storage (Hamidi Moghaddam et al. 2024; Hosseini et al. 2023). This is likely due to the conversion of lactose into lactic acid by starter cultures and the formation of ionic groups during proteolysis. In this context, similar findings have been reported by other researchers (Hala et al. 2010; Popescu et al. 2023).

**TABLE 2:** Changes in pH and titratable acidity of the processed cheese samples during 60 days of storage at 4°C.

	Samples	Storage time (Days)		
		0	30	60
pH	Control	5.81 ± 0.02^Aa^	5.67 ± 0.04^Ab^	5.47 ± 0.03^ABc^
	2% AE	5.82 ± 0.03^Aa^	5.67 ± 0.02^Ab^	5.46 ± 0.02^Bc^
	2% AENL	5.81 ± 0.05^Aa^	5.66 ± 0.05^Ab^	5.48 ± 0.07^ABc^
	10% AE	5.83 ± 0.05^Aa^	5.67 ± 0.05^Ab^	5.50 ± 0.10^ABc^
	10% AENL	5.86 ± 0.05^Aa^	5.69 ± 0.06^Ab^	5.53 ± 0.05^ABc^
	20% AE	5.86 ± 0.04^Aa^	5.68 ± 0.03^Ab^	5.55 ± 0.05^ABc^
	20% AENL	5.89 ± 0.06^Aa^	5.71 ± 0.04^Ab^	5.57 ± 0.06^Ac^
Titratable acidity (%LA)	Control	1.12 ± 0.08^Ac^	1.51 ± 0.01^Ab^	2.33 ± 0.06^Ba^
	2% AE	1.03 ± 0.06^ABc^	1.52 ± 0.11^Ab^	2.52 ± 0.02^Aa^
	2% AENL	1.10 ± 0.09^Ac^	1.60 ± 0.06^Ab^	2.22 ± 0.11^Ba^
	10% AE	0.96 ± 0.05^BCc^	1.53 ± 0.06^Ab^	1.93 ± 0.03^Ca^
	10% AENL	0.94 ± 0.06^BCc^	1.23 ± 0.13^Bb^	1.84 ± 0.10^Ca^
	20% AE	0.88 ± 0.08^Cc^	1.44 ± 0.12^Ab^	1.67 ± 0.07^Da^
	20% AENL	0.86 ± 0.07^Cb^	1.01 ± 0.09^Cb^	1.61 ± 0.10^Da^

*Note:* Different capital letters in each column indicate significant differences among treatments and different small letters in each row indicate significant differences in storage days (*p* < 0.05).

At the end of storage time, the pH and acidity values of the processed cheese varied between 5.47 ± 0.03, 2.33 ± 0.06 for the control sample and 5.57 ± 0.06, 1.61 ± 0.10 for the sample containing 20% AENL, respectively. As it is clear from the obtained data, the highest amount of pH reduction was related to the control sample. Because the addition of AE to process cheese prevents the post‐fermentation process during storage to a great extent, the decrease in pH was lower in fortified cheeses. This occurrence shows the high potential of AE in preserving cheese and inhibiting the growth of microorganisms during storage. In agreement with our study, Azarashkan, Motamedzadegan, et al. (2022) stated that untreated cheese samples had lower pH values compared to samples enriched with nano‐encapsulated broccoli sprout extract. In another study, it was reported that the pH value of cream cheese samples containing dried curry leaves ( *Murraya koenigii* L.) powder was higher than the control sample (Weragama et al. 2021). However, in the research of Balabanova et al. (2020), the addition of encapsulated pepper ( *Capsicum annuum* ) extracts did not have a significant effect on the pH of labneh cheese samples.

#### 3.2.2 Total Phenolic Content (TPC)

Phenolic compounds are a broad group of phytochemicals with one or more phenol units in their structure and are naturally present in plant sources. These compounds are found in the aerial parts of plants, including seeds, leaves, flowers, and fruits (Hosseini et al. 2023). Today, due to their unique antioxidant properties, they have received much attention. The Total phenolic content of the AE used in this study was calculated as 1.6 ± 0.10 mg GAL/mL. As seen in Table 3, on the first day, the lowest value of total phenolic content was obtained for the control sample (65.60 ± 1.40 mg gallic acid/kg sample). As expected, TPC increased significantly with the addition of AE or AENL and increasing its concentration in PC samples. Phenolic compounds, including flavonoids (ferulic acid, chlorogenic acid, p‐hydroxybenzoic acid, coumaric acid, and caffeic acid) and phenolic acids (rutin, kaempferide, andquercetin), are the dominant bioactive compounds in asparagus (Chen et al. 2017).

**TABLE 3:** Changes in TPC and DPPH radical scavenging activity of the processed cheese samples during 60 days of storage at 4°C.

	Samples	Storage time (Days)		
		0	30	60
TPC (mg GAE/100 g sample)	Control	65.60 ± 1.40^Ga^	55.27 ± 0.57^Gb^	39.03 ± 0.08^Fc^
	2% AE	78.23 ± 101^Ea^	62.13 ± 0.93^Fb^	41.40 ± 1.53^Fc^
	2% AENL	72.35 ± 1.34^Fa^	69.26 ± 0.99^Ea^	61.44 ± 2.59^Eb^
	10% AE	123.16 ± 2.52^Da^	97.34 ± 0.68^Db^	78.26 ± 1.09^Dc^
	10% AENL	131.62 ± 2.60^Ca^	126.20 ± 2.00^Cb^	114.21 ± 2.42^Cc^
	20% AE	168.09 ± 1.24^Ba^	137.33 ± 2.39^Bb^	103.24 ± 3.32^Bc^
	20% AENL	175.48 ± 2.18^Aa^	158.41 ± 1.46^Ab^	145.11 ± 1.94^Ac^
DPPH radical scavenging activity (%)	Control	20.31 ± 0.70^Da^	18.43 ± 1.19^Eb^	16.26 ± 0.85^Ec^
	2% AE	22.34 ± 1.11^CDa^	21.33 ± 1.42^Da^	18.24 ± 1.34^DEb^
	2% AENL	24.17 ± 1.53^Ca^	23.25 ± 1.67^Da^	21.30 ± 1.36^Da^
	10% AE	34.53 ± 1.37^Ba^	31.59 ± 1.37^Cb^	28.47 ± 1.62^Cc^
	10% AENL	33.39 ± 2.34^Ba^	32.04 ± 1.03^Ca^	30.52 ± 0.44^BCa^
	20% AE	42.28 ± 2.41^Aa^	37.24 ± 0.72^Bb^	32.53 ± 0.50^Bc^
	20% AENL	44.06 ± 1.07^Aa^	42.45 ± 0.47^Aa^	39.51 ± 1.71^Ab^

*Note:* Different capital letters in each column indicate significant differences among treatments and different small letters in each row indicate significant differences in storage days (*p* < 0.05).

The phenolic compound content of cheese samples decreased during 2 months of storage, but this decrease was significantly less in cheeses containing AENL than in samples containing non‐encapsulated extract (Table 3). The highest reduction was for the control sample and the sample with 2% AENL (47% and 40%, respectively), and the lowest reduction was for the cheese sample with 10% encapsulated asparagus extract (12%). The decrease in TPC of cheese samples during storage is attributed to the susceptibility of free phenolic compounds to oxidation and degradation under the influence of oxygen, heat, light, and LAB over time (Azarashkan, Motamedzadegan, et al. 2022; Hamidi Moghaddam et al. 2024). The nanoencapsulation of the extract improved the preservation of phenolic compounds and enabled their controlled release. Zou et al. (2014) also concluded in their study that encapsulating tea polyphenols in nanoliposomes increased their stability. In addition, other researchers have shown that encapsulation can preserve the stability of olive (Farrag et al. 2020) and chia ( *Salvia hispanica* L.) seed extract (Hosseini et al. 2023) phenolic compounds in cheese samples during storage.

#### 3.2.3 Antioxidant Activity

One of the prominent roles of antioxidants is to scavenge free radicals. Because these compounds, through the oxidation of proteins, lipids, and DNA, lead to the production of toxic compounds and disruption of the functioning of biological systems and membranes. Therefore, they play a critical role in preventing various diseases and maintaining health (Kou et al. 2015). DPPH radical is a stable free radical that is mainly utilized to investigate the antioxidant activity of compounds. DPPH radical scavenging method is considered the simplest and at the same time the most sensitive spectrophotometric assay for determining the antioxidant activity of plant extracts (Azarashkan, Motamedzadegan, et al. 2022). The antioxidant activity of the AE was obtained at 48.56% ± 0.93%. As seen in Table 3, the amount of antioxidant activity of cheese samples increases with increasing the concentration of asparagus extract (in free or nanoliposome form), which is obviously due to the increase in the TPC of the cheese samples. According to the report of Chen et al. (2017), there is a direct and positive relationship between the phenolic content (especially rutin) and the antioxidant activity of asparagus. However, throughout the storage time, the antioxidant activity decreased in all samples. The reduction pattern of antioxidant activity of cheese samples was almost similar to the reduction pattern of TPC. So, the highest reduction was related to the control sample and the sample with 2% AE (40% and 33%, respectively) and the lowest reduction was related to the cheese sample containing 10% of AENL (8%). The decline in the DPPH radical scavenging percentage, which reflects a reduction in antioxidant activity over time, is likely attributed to enhanced interactions between proteins and phenols (Haseli et al. 2023). It has been stated that phenolic compounds interact with proteins via various mechanisms, such as hydrophobic interactions, hydrogen bonds, covalent bonds, and ionic bonds. These interactions can modify the activity of polyphenolic compounds in cheese. Additionally, polyphenols exhibit stronger antioxidant effects in their free form compared to when they are bound to proteins (Mousavi et al. 2023).

As previously mentioned, the encapsulation of extract leads to higher stability of phenolic compounds and consequently maintenance of antioxidant activity in treated cheeses during storage. Similar results have been reported by other researchers (Azarashkan, Farahani, et al. 2022; Pasini Deolindo et al. 2019; Pérez‐Soto et al. 2021). Based on the study of Gortzi et al. (2008) regarding the extract of *Myrtus communis* , an increase in antimicrobial and antioxidant activity was observed after liposome encapsulation, so that its antioxidant activity was 25% higher than the pure extract. Also, an increase in the stability of the antioxidant activity of mangosteen ( *Garcinia mangostana* L.) peel extract (Indiarto et al. 2023), proanthocyanidins and α‐tocopherol (Zhao et al. 2024) and avocado seed extract (Chuacharoen et al. 2024) has been reported as a result of encapsulation. This issue can be due to encapsulation protection against hydrolysis and oxidation conditions.

#### 3.2.4 Sensory

The results obtained from the sensory evaluation of PC samples containing different levels of AE and AENL during 2 months of storage at 4°C are shown in Table 4. On the first day, incorporating AENL up to a concentration of 10% and AE up to a concentration of 2% had no remarkable effect on the parameters of texture, flavor, color, odor, and overall acceptability. In terms of texture, the PC containing 20% AE or AENL was significantly different from the control sample (*p* < 0.05). In fact, high levels of extract (in free or encapsulated form) cause the texture to become too soft and undesirable for the consumer. The softening of the texture is potentially due to the increased moisture content (Hosseini et al. 2023). In line with our study, other researchers also found that the addition of microencapsulated chavil (*Ferulago angulata*) extract (Borhanpour et al. 2022), nanoemulsion and *Opuntia oligacantha* microcapsule (Pérez‐Soto et al. 2021), and oregano essential oil nanoemulsion (Artiga‐Artigas et al. 2017) to fresh cheese samples reduces their hardness. Also, with the incorporation of AE at 10% or 20% levels, the flavor, odor, and overall acceptability were greatly reduced (*p* < 0.05). This indicates that encapsulation can cover the negative effects of the extract on the sensory characteristics of cheese. Similar findings were obtained by Azarashkan, Motamedzadegan, et al. (2022), who found that adding free‐form broccoli sprout extract had a significant negative effect on the taste and overall acceptability of ricotta cheese samples.

**TABLE 4:** Changes in odor, color, flavor, texture, and overall acceptability of the processed cheese samples during 60 days of storage at 4°C.

	Samples	Storage time (Days)		
		0	30	60
Odor	Control	4.97 ± 0.06^Aa^	3.17 ± 0.21^Eb^	1.43 ± 0.15^Dc^
	2% AE	4.87 ± 0.06^Aa^	3.47 ± 0.15^DEb^	2.33 ± 0. 15^Cc^
	2% AENL	4.93 ± 0.12^Aa^	3.60 ± 0.10^Db^	2.47 ± 0.21^Cc^
	10% AE	4.30 ± 0.10^Ca^	3.97 ± 0.06^BCb^	3.57 ± 0. 25^Bc^
	10% AENL	4.83 ± 0.06^Aa^	4.37 ± 0.32^Ab^	4.10 ± 0. 10^Ab^
	20% AE	3.90 ± 0.10^Da^	3.77 ± 0.12^CDa^	3.63 ± 0.23^Ba^
	20% AENL	4.57 ± 0.06^Ba^	4.23 ± 0.15^ABb^	3.87 ± 0.06^ABc^
Color	Control	4.93 ± 0.06^Aa^	3.50 ± 0.10^Eb^	2.47 ± 0.25^Ec^
	2% AE	4.90 ± 0.10^Aa^	3.73 ± 0.12^Da^	2.90 ± 0.12^Db^
	2% AENL	4.90 ± 0.10^Aa^	3.77 ± 0.12^Da^	3.20 ± 0. 15^CDb^
	10% AE	4.70 ± 0.21^BCa^	4.10 ± 0.10^Ba^	4.00 ± 0.06^Ab^
	10% AENL	4.87 ± 0.06^ABa^	4.20 ± 0.15^ABa^	3.70 ± 0.32^ABb^
	20% AE	4.50 ± 0.10^Da^	3.96 ± 0.06^Ca^	3.50 ± 0.15^BCb^
	20% AENL	4.67 ± 0.15^CDa^	4.30 ± 0.00^Aa^	4.00 ± 0.10^Ab^
Flavor	Control	4.97 ± 0.06^Aa^	3.37 ± 0.15^Db^	2.07 ± 0.12^Dc^
	2% AE	4.83 ± 0.06^Aa^	3.73 ± 0.36^Cb^	2.93 ± 0.15^Cc^
	2% AENL	4.93 ± 0.06^Aa^	3.90 ± 0.10^BCa^	3.07 ± 0. 12^Cb^
	10% AE	4.07 ± 0.25^Ca^	4.17 ± 0.21^ABa^	3.50 ± 0.44^Bb^
	10% AENL	4.73 ± 0.06^ABa^	4.33 ± 0.25^Aa^	4.03 ± 0.25^Ab^
	20% AE	3.13 ± 0.15^Da^	2.97 ± 0.06^Ea^	2.83 ± 0.29^Cb^
	20% AENL	4.60 ± 0.10^Ba^	4.13 ± 0.15^ABa^	3.97 ± 0.06^Ab^
Texture	Control	4.93 ± 0.12^Aa^	3.13 ± 0.15^Db^	2.00 ± 0.10^Fc^
	2% AE	4.90 ± 0.00^Aa^	4.13 ± 0.12^ABb^	3.07 ± 0.21^Ec^
	2% AENL	4.93 ± 0.06^Aa^	4.13 ± 0.15^ABb^	3.63 ± 0.15^BCc^
	10% AE	4.60 ± 0.10^Ba^	3.93 ± 0.38^Ba^	3.83 ± 0.12^ABb^
	10% AENL	4.57 ± 0.21^Ba^	4.20 ± 0.20^Aab^	4.10 ± 0.20^Ab^
	20% AE	3.90 ± 0.17^Ca^	3.47 ± 0.06^Ca^	3.10 ± 0.17^DEa^
	20% AENL	3.80 ± 0.10^Ca^	3.67 ± 0.12^Cb^	3.37 ± 0.30^CDb^
Overall acceptability	Control	4.97 ± 0.06^Aa^	3.23 ± 0.21^Db^	1.90 ± 0.20^Dc^
	2% AE	4.80 ± 0.10^ABa^	3.20 ± 0.10^Db^	3.03 ± 0.06^Cb^
	2% AENL	4.93 ± 0.06^Aa^	3.47 ± 0.25^CDb^	3.30 ± 0.35^Cb^
	10% AE	4.57 ± 0.15^CDa^	3.90 ± 0.17^ABb^	3.63 ± 0.15^Bb^
	10% AENL	4.70 ± 0.10^BCa^	4.13 ± 0.28^Ab^	4.00 ± 0.10^Ab^
	20% AE	3.97 ± 0.15^Ea^	3.73 ± 0.15^BCa^	3.20 ± 0.26^Cb^
	20% AENL	4.43 ± 0.15^Da^	4.03 ± 0.12^ABb^	3.77 ± 0.15^ABb^

*Note:* Different capital letters in each column indicate significant differences among treatments and different small letters in each row indicate significant differences in storage days (*p* < 0.05).

During storage, the sensory acceptability of all samples decreased due to microbial growth and chemical reactions. However, the most significant reduction was obtained for the control sample and the sample containing 2% AE. The decrease in sensory rating of samples containing free extract is due to the decomposition or oxidation of its components (Al‐subhi 2025). High concentrations of asparagus extract can delay the destructive reactions in cheese and thus maintain their sensory quality throughout refrigerated storage. Since encapsulation can protect bioactive substances and improve their distribution in food products (Soliman, Negm El‐Dein, et al. 2024), the sensory quality of the cheese samples fortified with AENL was favorable until the 60th day of cold storage. In the study conducted by El‐Den (2020), adding curcumin improved the sensory acceptance of ricotta cheese samples during storage. The enrichment of soft cheese with rosemary extract did not have a significant effect on its taste, texture, appearance, and overall acceptability (Hala et al. 2010). Balabanova et al. (2020) reported that the sensory quality of labneh cheese containing encapsulated pepper extract is maintained during storage. Farrag et al. (2020) stated that although the incorporation of olive polyphenol extract into soft white cheese formulation did not significantly affect the sensory characteristics of the sample, adding encapsulated polyphenol extract olive notably improved the overall acceptance of samples. Besides, the addition of mandarin peel extract nanoliposomes has no adverse effect on the sensory properties of processed cheese (El‐Messery et al. 2019).

## 4 Conclusion

The findings obtained from this research showed that asparagus extract can be successfully encapsulated inside nanoliposomes and applied to enrich processed cheese. The incorporation of asparagus extract in the cheese prevented excessive pH reduction during cold storage, which can be attributed to its protective and antimicrobial properties. The nanoencapsulation of AE with nanoliposomes has a positive effect on the stability of the bioactive and phenolic compounds of the extract and thus maintains the antioxidant activity of the samples throughout 60 days of storage. In this study, a direct relationship was shown between total phenol content and antioxidant activity. Finally, it can be said that with the successful production of functional processed cheese using AENL, as a rich source of bioactive compounds, we can take a big step towards producing functional PC with health benefits. Therefore, the results obtained in this research provide new insights for developing natural products for scientists and food manufacturers. However, the analysis of the physicochemical properties of asparagus extract, texture profile, and the effect of adding asparagus extract to other food products should be considered in future research.

## Author Contributions

**Parisa Solhi:** methodology (equal), resources (equal), writing – original draft (equal). **Mahdieh Salari:** data curation (equal), investigation (equal), writing – review and editing (lead). **Hamed Hamishehkar:** conceptualization (equal), project administration (lead), supervision (lead).

## Ethics Statement

The authors have nothing to report.

## Consent

Written informed consent was obtained from all participants in the study.

## Conflicts of Interest

The authors declare no conflicts of interest.

## Data Availability

All the data used are presented in this article.
